# Wasabi (*Eutrema japonicum*) Reduces Obesity and Blood Pressure in Diet-Induced Metabolic Syndrome in Rats

**DOI:** 10.3390/foods11213435

**Published:** 2022-10-29

**Authors:** Fernanda Santos Thomaz, Yuen P. Tan, Craig M. Williams, Leigh C. Ward, Simon Worrall, Sunil K. Panchal

**Affiliations:** 1 School of Chemistry and Molecular Biosciences, The University of Queensland, Brisbane, QLD 4072, Australia; 2 Functional Foods Research Group, University of Southern Queensland, Toowoomba, QLD 4350, Australia; 3 School of Science, Western Sydney University, Richmond, NSW 2753, Australia; 4 Global Centre for Land-Based Innovation, Western Sydney University, Richmond, NSW 2753, Australia

**Keywords:** wasabi, glucosinolate, 6-(methylsulfinyl)hexyl isothiocyanate, obesity, metabolic syndrome

## Abstract

6-(Methylsulfinyl)hexyl isothiocyanate (6-MSITC) has several biological functions. The present study aimed to evaluate the composition of hydroponically grown Tasmanian wasabi (*Eutrema japonicum* (Miq.) Koidz.) for 6-MSITC in all plant tissues and investigate the influence of wasabi (rhizome and stem blend) in high-carbohydrate, high-fat (H) diet-fed rats. Male Wistar rats were fed either a corn starch (C) or H diet. After the initial 8 weeks, half of the animals on the C and H diets were given 5% (*w/w*) wasabi powder in their respective diets for an 8-week duration (CW and HW). The control animals received diets without supplementation throughout the 16-week experiment. Our findings demonstrated that wasabi grown under hydroponic conditions contained 6-MSITC in all parts of the plant such as the stem, leaf and flower, as well as the commonly used rhizome, albeit at lower concentrations. Rats treated with wasabi showed reductions in body weight (H, 460.0 ± 9.5; HW, 416.0 ± 3.6 g), fat mass (H, 178 ± 14; HW, 120 ± 23 g), plasma triglycerides (H, 1.7 ± 0.3; HW, 0.9 ± 0.3 mmol/L) and total cholesterol (H, 1.5 ± 0.1; HW, 1.0 ± 0.04 mmol/L), and the plasma activities of aspartate transaminase. Systolic blood pressure and the area under the curve of blood glucose concentration were decreased by wasabi treatment. Thus, wasabi may be a novel alternative treatment to assist in the management of obesity and related metabolic disorders.

## 1. Introduction

The prevalence of obesity and metabolic syndrome has rapidly increased and is contributing to major public health problems worldwide [1]. This increase in prevalence is associated with an augmented risk of developing cardiovascular disease, type 2 diabetes, non-alcoholic fatty liver disease and certain cancers [1]. Functional foods can play an important role in the treatment of obesity and metabolic syndrome [2]. The concept and integration of functional foods as natural supplements is continually increasing worldwide. However, the absence of clinical trials and strong scientific evidence for many functional foods including wasabi is an obstacle in using these products in traditional medicine [2].

**Figure 1:**
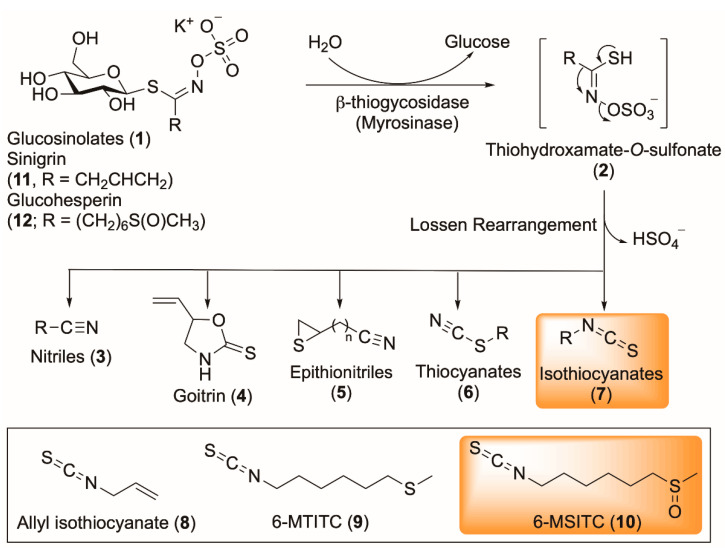
Myrosinase-catalysed hydrolysis of wasabi glucosinolates.

**Figure 2:**
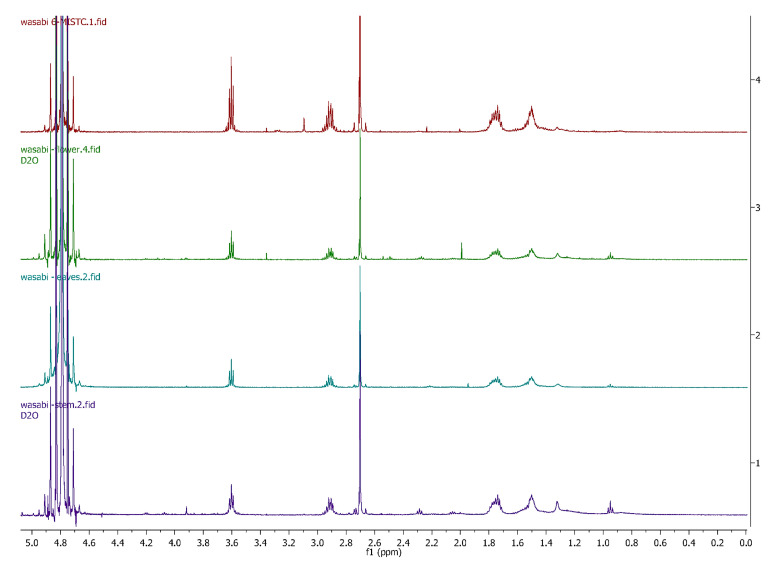
^1^H NMR of 6-MSITC compared in *E. japonicum* flower, leaves and stem recorded in D_2_O.

**Figure 3:**
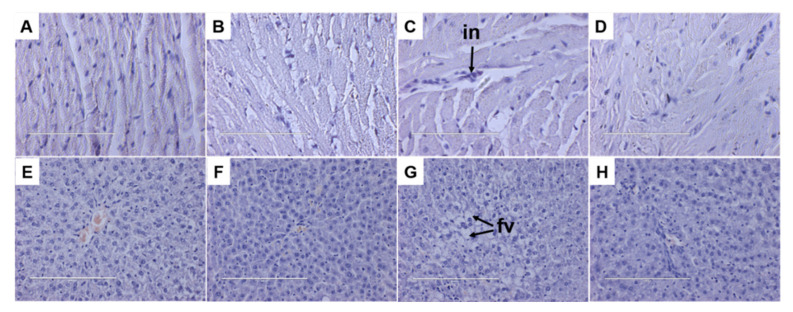
Effects of wasabi on heart inflammation and fat deposition in the liver. (**A**–**D**) Hematoxylin and eosin staining (40×) of the left ventricle showing infiltration of inflammatory cells (in) and (**E**–**H**) hematoxylin and eosin staining of the liver showing fat vacuoles (fv) from corn starch diet-fed rats (**A**,**E**), corn starch diet-fed rats supplemented with wasabi (**B**,**F**), high-carbohydrate, high-fat diet-fed rats (**C**,**G**) and high-carbohydrate, high-fat diet-fed rats supplemented with wasabi (**D**,**H**).

Wasabi is a member of the *Brassicaceae* family. The *Brassicaceae* (or *Cruciferae*) family of plants, including wasabi, contain glucosinolates (Figure 1—(**1**)) [3], that break down to give the distinctive pungent and bitter taste experienced by the consumer [4]. Although glucosinolates are generally considered benign, they readily undergo hydrolysis through the action of plant tissue mastication or chopping, which releases the enzyme myrosinase (β-thioglucosidase) [5]. The hydrolysis process, which generates thiohydroxamate-O-sulfonate (Figure 1—(**2**)), is followed by a Lossen-type rearrangement or other closely related 1,2-shifts, resulting in the production of nitriles (Figure 1—(**3**)), goitrin (Figure 1—(**4**)), epithionitriles (Figure 1—(**5**)), thiocyanates (Figure 1—(**6**)) and isothiocyanates (Figure 1—(**7**)), all depending on pH and substitution pattern (i.e., the R group) [3,4,5].

The isothiocyanates, which are a major product of the myrosinase-glucosinolate system, have been a focus of research efforts, especially in terms of understanding the health benefits of wasabi and associated biological activities [6,7]. Isothiocyanates are the main constituent of wasabi, particularly allyl isothiocyanate (AITC, Figure 1—(**8**)) (around 100–150 mg/100 g), along with lesser amounts of 6-(methylthio)hexyl isothiocyanate (6-MTITC, Figure 1—(**9**)), and 6-(methylsulfinyl)hexyl isothiocyanate (6-MSITC, Figure 1—(**10**)). The latter being derived from sinigrin (Figure 1—(**11**)) and glucohesperin (Figure 1—(**12**)), respectively [8,9]. 6-MSITC has been shown to suppress the activities of cyclooxygenase-2 and inducible nitric oxide synthase, and lipopolysaccharide-induced cytokine production including the inflammatory cytokines tumour necrosis factor, interferon-γ and the interleukin (IL)-1β and IL-6 [10]. Wasabi extract has previously demonstrated anti-obesogenic effects in high-fat diet-fed mice, and antiplatelet, antibacterial, antimutagenic and anticancer activity in the human carcinogenic cell lines in vitro [11]. Wasabi leaf extract is reported to decrease the concentrations of circulating triglycerides and total cholesterol, and suppress the gene expression of peroxisome proliferator-activated receptor γ (PPAR-γ) and sterol regulatory element-binding protein 1c (SREBP 1c) in obese mice; as well as improving β-oxidation of fatty acids, and modulating blood leptin and glucose concentrations [12]. While wasabi and its components have been studied in vitro, there is limited information on the influence of wasabi and its components on metabolic syndrome in vivo.

Considering, that 6-MSITC shows a range of promising biological activities, and that Australian-grown wasabi has yet to be investigated for 6-MSITC content, we determined the content of 6-MSITC being produced by hydroponically grown Tasmanian wasabi (*Eutrema japonicum* (Miq.) Koidz., previously known as *Wasabia japonica*) stem and rhizome blend. In addition, we evaluated the effectiveness of 6-MSITC in affecting obesity and related biomarkers, including body composition, blood glucose, systolic blood pressure, and heart and liver structural changes in a Wistar rat model of obesity and metabolic syndrome.

## 2. Materials and Methods

### 2.1. Plant Material

The hydroponic wasabi (*Eutrema japonicum* (Green thumb)) rhizome and stem blend powder was purchased from Shima Wasabi Pty Ltd. (Launceston, TAS, Australia; Figure S1) and synthetic pure 6-MSITC was purchased from Abcam (Cambridge, UK). The leaves, flowers and stems were kindly provided by Shima Wasabi Pty Ltd.

### 2.2. Composition of Wasabi Powder (Stem and Rhizome)

Phytochemical properties of samples were analysed for moisture content, ash, protein, total carbohydrate and fat. All the determinations were undertaken in triplicate and the results were expressed as the average value. The moisture content was calculated as loss in weight of the original sample after heating at 105 °C [13]. The overall protein concentration was calculated according to the Association of Official Agricultural Chemist’s official methods of analysis, using a nitrogen factor of 6.25. Crude fibre was estimated by acid/alkaline hydrolysis of insoluble residues, AOAC Official Method 962.09 [14]. Lipid extractions were performed using the Soxhlet extraction method [15], and elemental composition was determined using a flame photometer and atomic absorption spectrophotometer, Perkin-Elmer Model 303, School of Agriculture and Food Sciences, The University of Queensland, Gatton, QLD, Australia. Measurements were made at a wavelength of 422.7 nm.

Wasabi powder (50 g) was rehydrated with Milli-Q water (250 mL), extracted with *n*-hexane (250 mL) and diethyl ether (250 mL) at room temperature for 40 min. The diethyl ether layer was washed with saline solution (200 mL), dried with anhydrous sodium sulphate, filtered and evaporated using a rotary evaporator to produce a crude extract. At this point, the crude extract was compared to an authentic standard of 6-MSITC, along with a second diethyl ether extract of the wasabi powder, using reverse-phase high-performance liquid chromatography (HPLC). The HPLC analysis was performed on an Agilent 1100 series equipped with an autosampler and UV detector at 220 nm. A Grace Prevail C18 column (5 μm; 25 × 2.1 mm) at 37 °C was used for all analyses. The mobile phase consisted of methanol and water (30:70 *v*/*v*; flow rate, 1.0 mL/min). The injection volume was 1 μL. All sample solutions were filtered through a 0.45 μm membrane filter prior to the HPLC analysis. Using this method, a retention time of 21.91 ± 0.03 minutes was observed for 6-MSITC (Figure S2). This was confirmed using ultra-high-performance liquid chromatography (uHPLC) (Figure S3). The crude extract was then subjected to gel-filtration chromatography using a Sephadex G-25 column (Φ 1.5 × 16 cm column (Cytiva, Tokyo, Japan); ethyl acetate:methanol:water 50:3:2 by volume) and fractionated into five fractions (10 mL of each fraction). Each fraction was subsequently analysed using uHPLC to identify the fraction containing 6-MSITC. The uHPLC analysis was performed on an Agilent 6540 TOF Electrospray Quadrupole-quadrupole-Time of Flight. An Agilent ZORBAX Eclipse Plus C18 column (1.8 μm; 2.1 × 100 mm) was used for analyses. The mobile phase consisted of 0.1% formic acid in water and 0.1% formic acid in acetonitrile (flow rate 1.0 mL/min).

^1^H NMR spectra were acquired on Bruker Avance 500 spectrometer at 298 K. ^13^C NMR spectra were recorded at 125 MHz. Coupling constants are given in hertz (Hz) and chemical shifts are reported as δ values in parts-per-million with the solvent resonance as the internal standard (^1^H NMR-CDCl_3_: δ 7.26 and D_2_O: δ 4.79). The separation process was conducted using Merck silica gel 60 (230–400 mesh). Thin layer chromatography (TLC) was performed on TLC plate silica gel 60 F254 0.2 mm (Merck, Darmstadt, Germany). Total fractions used in the NMR analysis were obtained at concentration of 1 mg/mL. Both uHPLC and MS/MS mass spectrometry were performed using an Agilent 6540 TOF Electrospray Quadrupole-quadrupole-Time of Flight at a low mass range (180–500). The 6-MSITC content in each sample was quantified by plotting peak areas into a standard curve (Figure S4). The sample concentrations and recoveries of 6-MSITC in wasabi are presented in Table S1.

### 2.3. Rats and Diets

All experimental protocols were approved by the Animal Ethics Committees of the University of Southern Queensland and The University of Queensland, under the guidelines of the National Health and Medical Research Council of Australia. Forty-eight male Wistar rats (eight to nine weeks old) weighing 330–340 g were sourced from the Animal Resource Centre, Murdoch, WA, Australia and were individually housed at 22 ± 2 °C with a 12 h light/dark cycle. Rats were randomly divided into four groups: corn starch diet-fed rats (C; *n* = 12); corn starch diet-fed rats supplemented with 5% wasabi powder (CW; *n* = 12); high-carbohydrate, high-fat diet-fed rats (H; *n* =12); and high-carbohydrate, high-fat diet-fed rats supplemented with 5% wasabi powder (HW; *n* =12). CW and HW rats were given C and H diets, respectively, for the first eight weeks without supplementation. Their diets were supplemented with 5% wasabi powder for the last eight weeks of the protocol. C and H rats were fed their diets for 16 weeks [16]. The composition of C and H diets have been described previously [16]. H and HW rats were given 25% fructose (*w/v*) in their drinking water while the C and CW rats received normal drinking water. Rats had free access to food and water throughout the protocol. The intakes of food and water and body weights were measured daily, and energy intake and feed efficiency were calculated [16].

### 2.4. Body Composition

Body composition in each group in week 16 was measured using Dual-energy X-ray absorptiometry as previously described [16]. Abdominal circumference was measured under light anesthesia as previously described [16].

### 2.5. Oral Glucose Tolerance and Systolic Blood Pressure Measurement

At the end of the protocol, rats were deprived of food overnight for 12 h. Basal blood glucose concentrations were measured in blood taken from the tail vein using a Medisense Precision Q.I.D. glucose meter (Abbott Laboratories, Bedford, MA, USA) as previously described. Blood samples were taken at 30, 60, 90 and 120 min after glucose administration [16]. Systolic blood pressure was measured in all rats under light sedation as previously described [16].

### 2.6. Plasma Biomarkers, Tissue Weights and Histology of Liver and Heart

Following euthanasia with intraperitoneal Lethabarb (pentobarbitone sodium, 100 mg/kg; Virbac, Peakhurst, NSW, Australia), heparin (~200 IU) was injected into right femoral vein before blood collection, centrifugation and plasma isolation. This plasma was used for measuring aspartate transaminase and alanine transaminase activities and concentrations of triglycerides and total cholesterol. Following blood collection, organ weights were measured for right and left ventricles, liver and retroperitoneal, epididymal and omental fat. These weights were normalised relative to the tibial length at the time of their removal (in mg/mm) [16]. The heart and liver were removed and fixed in 10% neutral buffered formalin solution for histopathological analysis [16]. Standard histological procedures were followed to process tissues for staining with hematoxylin and eosin [16]. The tissue was imaged using EVOS FL Colour Imaging System (version 1.4 (Rev 26059); Advanced Microscopy Group, Bothwell, WA, USA).

### 2.7. Statistical Analysis

All data are presented as mean ± standard error of the mean (SEM). The variables that were not normally distributed were transformed (using the log10 function) before statistical analysis. When equality of variances was indicated by a Bartlett’s test, statistical analysis was performed using a two-way analysis of variances to test for the effects of diets, treatment and/or their interactions. Multiple comparison post-test analysis was done using Newman-Keuls analysis when the interaction and/or the main effects were significant (*p* < 0.05). All statistical analyses were performed using GraphPad Prism version 5.0 for Windows (GraphPad, San Diego, CA, USA).

## 3. Results and Discussion

### 3.1. Composition of Wasabi Powder

Dried wasabi contained following proportions of nutrients: carbohydrate, 76.6%; protein, 1.78 ± 0.03%, lipid, 0.96 ± 0.01%; minerals, 9.25 ± 0.04%. The mineral content included: Sulphur, 7.66 ± 0.08 mg/g; calcium, 5.76 ± 0.05 mg/g; magnesium, 1.33 ± 0.01 mg/g; phosphorus, 3.09 ± 0.03 mg/g; potassium, 32.02 ± 0.59 mg/g; and sodium, 0.08 ± 0.01 mg/g (Table 1).

Wasabi rhizome dry powder contained 7.1 mg/g dry weight of 6-MSITC (Table S1). This value was 17% higher than those in fresh Japanese or Korean wasabi leaves, stems or roots [17], though this is probably due to the reduced water content in the dry powder. Another study determined ~550–556 μg/g of 6-MSITC in wet weight of wasabi root [10]. The present study observed a concentration of 120–150 μg/g wet weight of 6-MSITC in stem and rhizome blend.

The 6-MSITC in the wasabi rhizome was obtained as a green-coloured oil. Its identity was confirmed via mass spectrometry and ^1^H and ^13^C NMR spectrometry compared against an authentic sample, in addition to matching literature data (Figures S3–S8; Tables S2–S4) [18,19]. Most of the volatile components in wasabi oils are *n*-alkenyl isothiocyanates and methylthioalkyl isothiocyanates, with AITC being the main component isolated [20]. 6-MSITC is reported to be much less volatile than AITC [8,21], but was readily obtained and handled.

### 3.2. Comparing Eutrema Japonicum Flower, Leaves and Stem

The ^1^H NMR spectra of wasabi (flower, leaf and stem) were overlayed showing good agreement that 6-MSITC was being extracted from these sections of the plant. The chromatographic repeatability was confirmed via triplicate injections of the diethyl ether-extracted material and demonstrated good inter-analysis reproducibility. The analysis of the wasabi plant material (flower, leaf and stem) showed high concentrations of 6-MSITC in the whole plant, with the stem having the greatest concentration of 6-MSITC, and the leaves the smallest concentrations (Figure 2).

### 3.3. Diet Intake, Body Composition and Metabolic Parameters

H rats gained more weight over 16 weeks compared to C rats (Table 2). Treatment with wasabi decreased body weight in both CW and HW rats compared to C and H rats, respectively (Table 2). Intakes of food and water were higher in C and CW rats compared to H and HW rats (Table 2). Energy intake was higher in C and CW rats compared to H and HW rats due to the higher energy content of H diet (Table 2). Abdominal circumference was higher in H rats compared to C rats and treated rats (Table 2). Diet or wasabi did not change whole-body lean mass in any of the groups. Whole-body fat mass was higher in H rats compared to C rats and HW rats had reduced whole-body fat mass compared to H rats (Table 2). Retroperitoneal, epididymal, omental and total abdominal fat were higher in H rats compared to C rats. Wasabi decreased retroperitoneal, epididymal, omental and total abdominal fat in HW rats compared to H rats (Table 2). Wasabi only decreased omental and total abdominal fat in CW rats compared C rats (Table 2).

Glucosinolates are a unique group of sulphur-containing secondary plant metabolites, and their hydrolysis products (isothiocyanates and sulphoraphanes) have been shown to modulate lipid metabolism [22]. Foods rich in isothiocyanates reduced body weight along with improved lipid profile in SHRSP/ZF rats [23]. Wasabi treated groups had a significant feed efficiency decline (Table 2). Among a variety of potential natural supplements for weight reduction are appetite suppressants. Natural plants such as *Caralluma fimbriata* and wasabi are natural appetite suppressants [24]. The appetite suppressing properties of wasabi have been attributed to wasabi’s pungent effect. Wasabi activated transient receptor potential ankyrin 1 (TRPA1) and transient receptor potential vanilloid 1 (TRPV1) [25,26,27]. Studies suggest that TRPA1 and TRPV1 may have a potential modulatory effect on ghrelin and/or glucagon-like peptide-1 secretion leading to reduced appetite [28,29]. Wasabi treatment also decreased the blood glucose area under the curve in CW and HW rats compared to C and H rats, respectively (Table 2). Wasabi’s main constituent, AITC, induced insulin secretion from pancreas via TRPV1 activation and increased carbohydrate oxidation in mice; these effects of allyl isothiocyanate were not observed in TRPV1-knockout mice, suggesting the role of TRPV1 in mediating responses of AITC [30,31]. Recently, role of TRPV1 and TRPA1 in attenuating obesity have been reviewed, suggesting that wasabi’s anti-obesity effects may have been mediated by these receptors [25]. Additionally, these receptors have been involved in upregulating UCP1 in brown fat, which is responsible for adaptive thermogenesis in response to cold adaptation and the diet [25]. Thermogenic changes in brown fat can be related to the UCP1 cascade leading to increased basal metabolism [32].

Plasma concentrations of total cholesterol and triglycerides were higher in H rats than in C rats (Table 2). Wasabi treatment in CW rats decreased plasma concentrations of total cholesterol without changing concentrations of triglycerides compared to C rats (Table 2). HW rats showed decreased plasma concentrations of total cholesterol and triglycerides compared to H rats.

Wasabi leaf extract reduced body weight gain, blood pressure and plasma triglyceride in animal studies [12,23]. Further, wasabi leaf extract inhibited adipose hypertrophy by suppressing PPARγ expression and stimulating the AMP-activate protein kinase (AMPK) activity by increased adiponectin [23]. In diabetic rats induced by high-fat diet and streptozotocin, allyl isothiocyanate reduced blood glucose, total cholesterol, triglycerides and creatinine concentrations, and increased total antioxidant capacity [12]. Allyl isothiocyanate administration increased expression of glucose transporter-2, PPARγ, insulin receptor substrate-1 and Nrf2 in the liver and kidney while downregulating NF-κB [12]. 5-Hydroxyferulic acid, another component from wasabi, has been shown to inhibit adipocyte differentiation in 3T3-L1 adipocytes [33].

Wasabi has been part of the diet in many populations, thus its safety is not an issue in developing it as a functional food or nutraceutical for metabolic syndrome. The dose used in this study corresponds to ~20–25 g daily intake of wasabi or ~220–250 mg daily intake of 6-MSITC for an adult human [34]. The daily dose of wasabi can be divided into smaller parts for inclusion in different meals of the day, thus, reducing the burden of consuming in a single dose. Another option would be to develop supplements containing 6-MSITC to provide this dose. Further, combination of different functional foods in the daily diet would assist in reducing the dose of each functional food item and increase the compliance by the general population.

### 3.4. Liver and Heart Parameters

Histological analysis of liver sections from the HW rats (Figure 3H) showed reduced fat deposition compared to H rats (Figure 3G). CW rats (Figure 3F) showed no changes in fat deposition in the liver compared to C rats (Figure 3E). In 3T3-L1 preadipocytes, AITC decreased lipid droplet accumulation in a dose-dependent manner, suppressed differentiation into adipocytes by decreasing galectin-12 expression and by downregulating key adipogenic transcription factors [35]. Further, in high-fat diet-fed mice, oral administration of AITC reduced body weight, lipid droplets in liver and white adipocyte size [35]. According to our results, dietary supplementation with wasabi reversed the increases in lipid profile induced by high-carbohydrate, high-fat diet. High-carbohydrate, high-fat diet leads to hepatic fat accumulation and liver dysfunction. Alanine transaminase and aspartate transaminase are biochemical markers commonly used in the diagnosis of liver damage [22]. Our results showed no changes in plasma alanine transaminase activity but reduced plasma aspartate transaminase activity (Table 2) suggestive of improved liver function.

Along with obesity, wasabi normalised the systolic blood pressure in HW rats. CW rats had the lowest systolic blood pressure among the groups (Table 2). There are various proposed mechanisms by which plant phytochemicals reduce blood pressure including vascular remodeling. Angiotensin II and ET-1 play an important role in vascular remodelling [36,37]. Wasabi reduced the plasma concentration of ET-1 in obese rats. Endothelin-1 is an important contributing factor in the development of hypertension in fructose-fed animals, due to its vasoconstrictor effect [38]. It is also a potential mediator of inducible nitric oxide synthase, inflammatory activities and fibrotic effects which can contribute to cardiovascular-related risks [39]. Nevertheless, further investigation is needed to identify the exact mechanism involved in the reduction of the blood pressure by wasabi.

Histology of the left ventricle showed increased infiltration of inflammatory cells in H rats (Figure 3C) compared to C rats (Figure 3A). This infiltration was reduced in left ventricle of HW rats (Figure 3D) with no changes in left ventricle of CW rats (Figure 3B). The reduction of inflammatory biomarkers by wasabi or 6-MSITC may be due to their modulatory effect of the Keap1-Nrf2 system [40,41,42,43] which is a major regulator of cellular responses to environmental stresses including lipopolysaccharide and pro-inflammatory cytokines such as IL-6 and IL-1β [44].

Wasabi extract has been shown to decrease inflammatory mediators such as cyclooxygenase-2, inducible nitric oxide synthase and cytokines such as IL-6, IL-1β, interferon γ and tumour necrosis factor [10,45,46], plasma adiponectin concentration, and expressions of acetyl-CoA carboxylase, fatty acid synthase, 3-hydroxy-3-methylglutaryl-coenzyme A reductase, CCAAT-enhancer-binding proteins, PPARγ and SREBP-1c in liver and adipose tissue [12]. This study has demonstrated a reduction of inflammatory cell infiltration in the heart of obese rats treated with wasabi. Allyl isothiocyanate and *Wasabia koreana* (a related species to wasabi) have shown anti-inflammatory effects in colitis and irritable bowel syndrome mice models [47,48,49]. Further, wasabi components including 6-MSITC have been shown to have anti-inflammatory effects through different mechanisms including inhibition of NF-κB pathway [10,47,50].

Allyl isothiocyanate is conjugated with glutathione or N-acetyl-cysteine and distributed in most organs and tissues [51]. Pharmacokinetic analysis showed rapid uptake and complete metabolism of allyl isothiocyanate following oral administration to rats suggesting that the potential benefits of wasabi may be observed due to the metabolites of allyl isothiocyanates such as 6-MSITC [51].

## 4. Conclusions

The study herein provides the first evidence that flowers, leaf and stem of Australian wasabi, as well as the commonly used rhizome, albeit at lower concentrations, contain 6-MSITC. The flowers had a higher concentration of 6-MSITC than the leaves but not the stems. Tasmanian wasabi attenuated hypertension and obesity in diet-induced metabolic syndrome in rats. It also attenuated the changes in acute inflammation in the heart and lipid deposition in the liver of Wistar rats. This study showed the potential of wasabi as an intervention for metabolic and physiological changes in obesity and related disorders, thus providing evidence to support the use of wasabi as a novel functional food. Confirming these outcomes through human intervention studies will assist in developing novel products with wasabi to reduce metabolic complications in society.

## Figures and Tables

**Table 1:** Composition of nutrients in hydroponic wasabi (*Eutrema japonicum*) rhizome and stem blend powder.

Composition	Value
Ash (%)	9.25 ± 0.04
Fat (%)	0.96 ± 0.01
Moisture (%)	11.30 ± 0.00
Carbohydrate (%)	71.5%
Dietary fibre (%)	5.1% ± 0.00
Protein (%)	1.78 ± 0.03
**Elements (mg/g)**	
Copper	0.03 ± 0.50
Calcium	5.76 ± 0.05
Iron	0.02 ± 0.00
Magnesium	1.33 ± 0.01
Manganese	0.01 ± 0.50
Phosphorus	3.09 ± 0.03
Potassium	32.02 ± 0.59
Sodium	0.08 ± 0.01
Sulphur	7.66 ± 0.08
Zinc	0.01 ± 0.00

**Table 2:** Effects of wasabi on physiological and metabolic parameters.

Variables	C	CW	H	HW	*p*-Value		
					Diet	Wasabi	Interaction
Water intake (mL/day)	40.6 ± 3.8 ^a^	40.0 ± 1.7 ^a^	33.3 ± 2.5 ^b^	29.5 ± 1.4 ^c^	0.74	0.34	0.4900
Food intake (g/day)	36.9 ± 1.3 ^a^	36.5 ± 2.6 ^a^	27.1 ± 2.1 ^b^	24.4 ± 0.9 ^c^	0.47	0.01	<0.0001
Energy intake (kJ/day)	413 ± 33 ^c^	410 ± 33 ^c^	553 ± 82 ^a^	484 ± 48 ^b^	0.47	0.0007	<0.0001
Feed efficiency (kJ/g)	0.15 ± 0.01 ^bc^	0.10 ± 0.01 ^c^	0.36 ± 0.03 ^a^	0.23 ± 0.02 ^b^	0.43	0.16	<0.0001
Body weight (g)	368 ± 5.3 ^a^	350 ± 4.2 ^b^	460 ± 9.5 ^c^	416 ± 3.6 ^d^	0.03	0.04	<0.0001
Abdominal circumference (cm)	18.3 ± 0.3 ^c^	17.8 ± 0.2 ^bc^	21.3 ± 1.0 ^a^	19.7 ± 0.3 ^b^	0.005	<0.0001	<0.0001
Whole-body lean mass (g)	289 ± 7 ^b^	289 ± 8 ^b^	318 ± 14 ^a^	315 ± 15 ^a^	0.88	0.25	0.0001
Whole-body fat mass (g)	89 ± 9 ^ab^	60 ± 6 ^c^	178 ± 14 ^a^	120 ± 23 ^b^	<0.0001	0.02	<0.0001
Wasabi intake (g/day)	-	1.7 ± 0.1 ^a^	-	1.0 ± 0.1 ^b^	-	-	-
Blood glucose area under the curve (mmol/L × min)	681 ± 24 ^b^	541 ± 27 ^c^	729 ± 34 ^a^	663 ± 20 ^b^	0.19	0.004	0.0006
Systolic blood pressure (mmHg)	131 ± 4 ^b^	128 ± 2 ^c^	146 ± 2 ^a^	132 ± 2 ^b^	<0.0001	0.0005	<0.0001
Plasma aspartate transaminase (U/L)	66.3 ± 3.7 ^b^	64.0 ± 4.0 ^c^	94.9 ± 10.4 ^a^	67.0 ± 5.0 ^b^	0.32	0.01	<0.0001
Plasma alanine transaminase (U/L)	26.1 ± 5.2 ^b^	24.0 ± 2.0 ^c^	37.9 ± 4.0 ^a^	37.0 ± 4.0 ^a^	0.39	0.43	<0.0001
Triglycerides (mmol/L)	0.5 ± 0.1 ^c^	0.5 ± 0.04 ^c^	1.7 ± 0.3 ^a^	0.9 ± 0.3 ^b^	0.37	0.02	<0.0001
Total cholesterol (mmol/L)	1.4 ± 0.1 ^a^	1.0 ± 0.1 ^b^	1.5 ± 0.1 ^a^	1.0 ± 0.04 ^b^	0.11	0.26	<0.0001
Retroperitoneal fat (mg/mm tibial length)	169 ± 19 ^b^	121 ± 6 ^c^	398 ± 67 ^a^	246 ± 24 ^b^	<0.0001	<0.0001	<0.0001
Epididymal fat (mg/mm tibial length)	90 ± 5 ^b^	75 ± 13 ^c^	172 ± 20 ^a^	123 ± 18 ^b^	<0.0001	<0.0001	<0.0001
Omental fat (mg/mm tibial length)	131 ± 10 ^b^	85 ± 6 ^c^	220 ± 13 ^a^	161 ± 20 ^b^	<0.0001	<0.0001	<0.0001
Total abdominal fat (mg/mm tibial length)	390 ± 20 ^ab^	281 ± 20 ^c^	790 ± 97 ^a^	530 ± 58 ^b^	<0.0001	<0.0001	<0.0001

Values are presented as mean ± SEM (*n* = 8–12). Means in a row with unlike superscripts (^a^, ^b^, ^c^ or ^d^) differ and no superscript indicates no significant difference between the groups, *p* < 0.05. C, corn starch diet-fed rats; CW, corn starch diet-fed rats supplemented with wasabi; H, high-carbohydrate, high-fat diet-fed rats; HW, high-carbohydrate, high-fat diet-fed rats supplemented with wasabi.

## Data Availability

The original data generated for the study are included in this article.

## Supplementary Materials

The following supporting information can be downloaded at: https://www.mdpi.com/article/10.3390/foods11213435/s1, Figure S1: Plant sections of Australian hydroponic wasabi: (A) flower; (B) leaf; (C) stem; (D) powder; (E) Shima Wasabi—Northern Tasmania Farm; Figure S2: Expanded chromatograms of the analysis of 6-MSITC by ultra-high performance liquid chromatography; Figure S3: uHPLC MS comparison of synthetic 6-MSITC (orange line) and that extracted (blue line) from *E. japonicum*; Figure S4: Standard curve of 6-MSITC. Results presented by plotting peak areas into a standard curve (r^2^ > 0.99); Figure S5: MS/MS spectra of *Eutrema japonicum* rhizome sample derived from n-hexane/diethyl ether extraction; Figure S6: ^1^H NMR comparison for authentic 6-MSITC (Top) and isolated 6-MSITC (bottom) from *E. japonicum* recorded in CDCl_3_; Figure S7: The ^1^H NMR spectra of 6-MSITC extracted from wasabi rhizome and stems; Figure S8: ^13^C NMR of the isolated 6-MSITC recorded in D_2_O at 125 MHz; Table S1: 6-MSITC concentration in hydroponic wasabi (*E. japonicum*) rhizome and stem blend powder; Table S2: Peak assignment identified the fragmentation pattern of 6-MSITC; Table S3: Tabulated ^1^H NMR data for 6-MSITC in D_2_O at 500 MHz; Table S4: ^13^C NMR data for 6-MSITC in D_2_O at 125 MHz.
