# Supplementary material for: Wasabi (Eutrema japonicum) Reduces Obesity and Blood Pressure in Diet-Induced Metabolic Syndrome in Rats
Source: Foods. 2022 Oct 29;11(21):3435. doi: 10.3390/foods11213435 (PMC9654917; doi:10.3390/foods11213435)
Supplement: Supplementary file 1 [file foods-11-03435-s001.zip › foods-1923948-supplementary.pdf]

foods

Article

# Wasabi (*Eutrema japonicum*) Reduces Obesity and Blood Pressure in Diet-Induced Metabolic Syndrome in Rats

**Fernanda Santos Thomaz [1,2], Yuen P. Tan [1], Craig M. Williams [1], Leigh C. Ward [1], Simon Worrall [1] and Sunil K. Panchal [2,3,4,*]**

[1] School of Chemistry and Molecular Biosciences, The University of Queensland, Brisbane, QLD 4072, Australia
[2] Functional Foods Research Group, University of Southern Queensland, Toowoomba, QLD 4350, Australia
[3] School of Science, Western Sydney University, Richmond, NSW 2753, Australia
[4] Global Centre for Land-Based Innovation, Western Sydney University, Richmond, NSW 2753, Australia
[*] Correspondence: s.panchal@westernsydney.edu.au; Tel.: +61-2-4570-1932

**Citation:** Thomaz, F.S.; Tan, Y.P.; Williams, C.M.; Ward, L.C.; Worrall, S.; Panchal, S.K. Wasabi (*Eutrema japonicum*) Reduces Obesity and Blood Pressure in Diet-Induced Metabolic Syndrome in Rats. *Foods* **2022**, *11*, 3435. https://doi.org/10.3390/foods11213435

Academic Editor: Kyung-Jin Yeum

Received: 2 September 2022
Accepted: 26 October 2022
Published: 29 October 2022

**Publisher's Note:** MDPI stays neutral with regard to jurisdictional claims in published maps and institutional affiliations.

**Copyright:** © 2022 by the authors. Licensee MDPI, Basel, Switzerland. This article is an open access article distributed under the terms and conditions of the Creative Commons Attribution (CC BY) license (https://creativecommons.org/licenses/by/4.0/).

## Supplementary Information

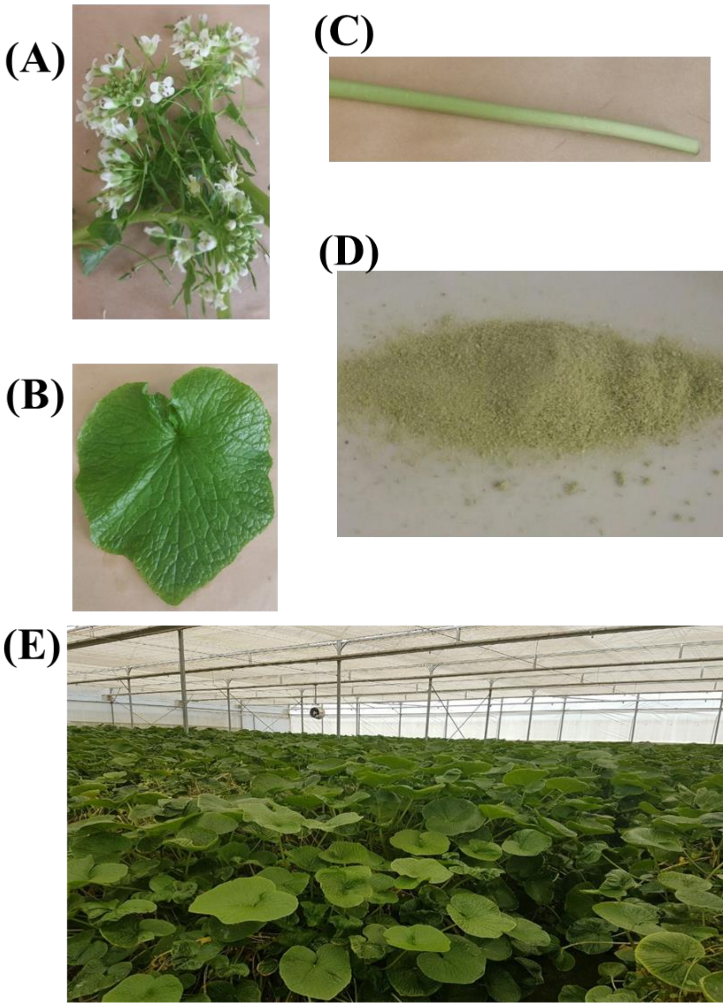


**Figure S1.** Plant sections of Australian hydroponic wasabi: (A) flower; (B) leaf; (C) stem; (D) powder; (E) Shima Wasabi - Northern Tasmania Farm.

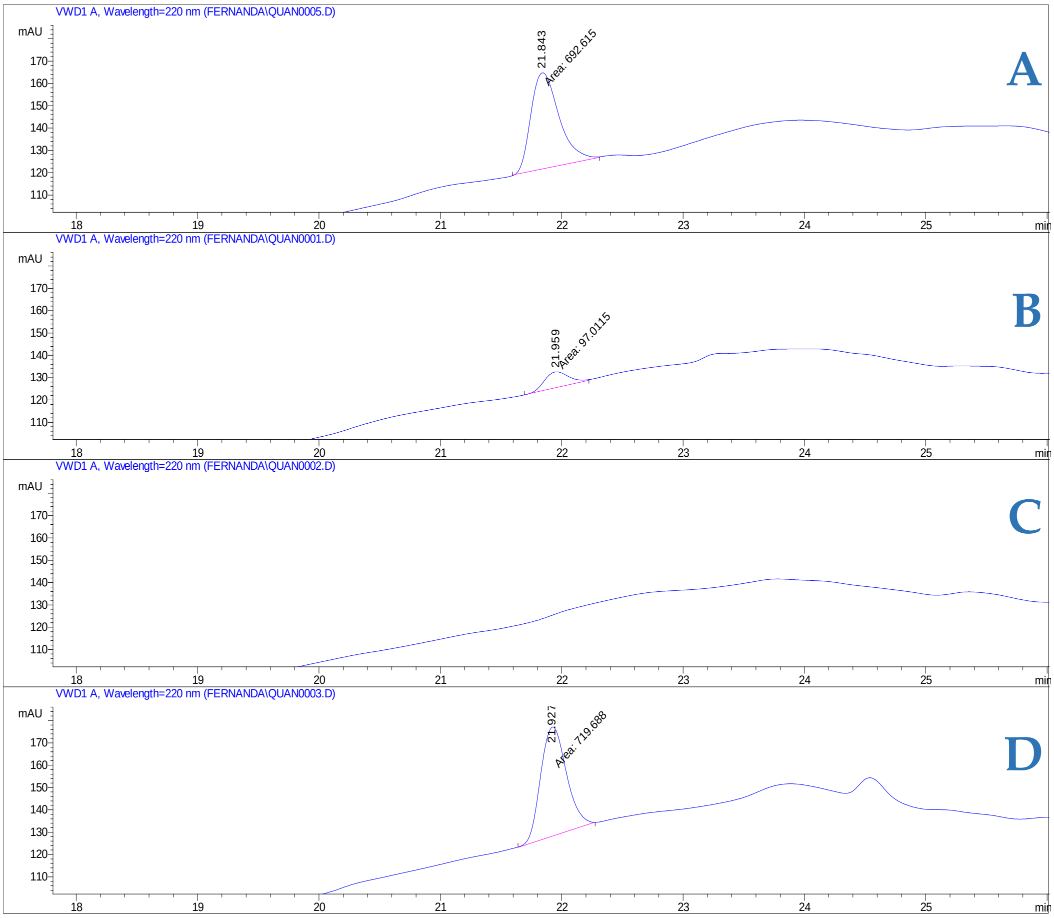


**Figure S2.** Expanded chromatograms of the analysis of 6-MSITC by ultra-high performance liquid chromatography. (A) Chromatogram of 5 mg/mL standard 6-MSITC, (B) Secondary ether extracted material, (C) Residual material after double ether extraction and (D) Primary 6-MSITC ether extraction.

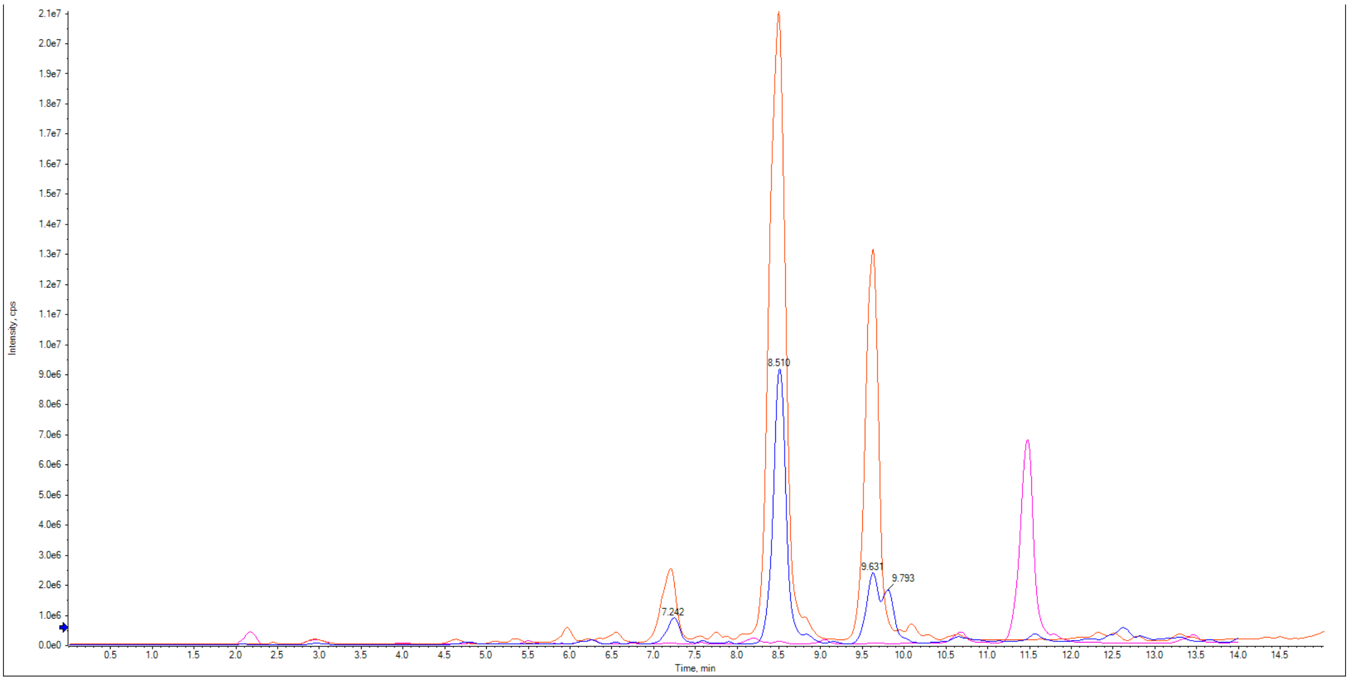


**Figure S3.** uHPLC MS comparison of synthetic 6-MSITC (orange line) and that extracted (blue line) from *E. japonicum*. The pink line shows the fraction containing only residual material from wasabi after the extraction, showing no detectable 6-MSITC.

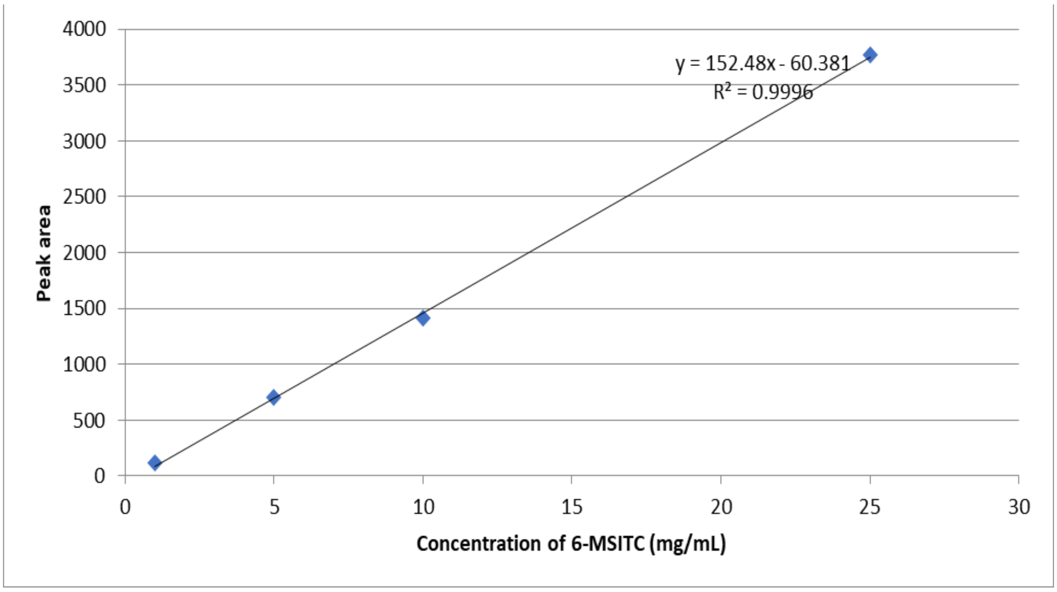


**Figure S4.** Standard curve of 6-MSITC. Results presented by plotting peak areas into a standard curve ($r^2 > 0.99$).

**Table S1.** 6-MSITC concentration in hydroponic wasabi (*E. japonicum*) rhizome and stem blend powder.

| Sample name | Concentration 6-MSITC (mg/mL) |
|---|---|
| Primary ether extracted material | 5.26 |
| Secondary ether extracted material | 1.85 |
| Residual material after double ether extraction | None detected |

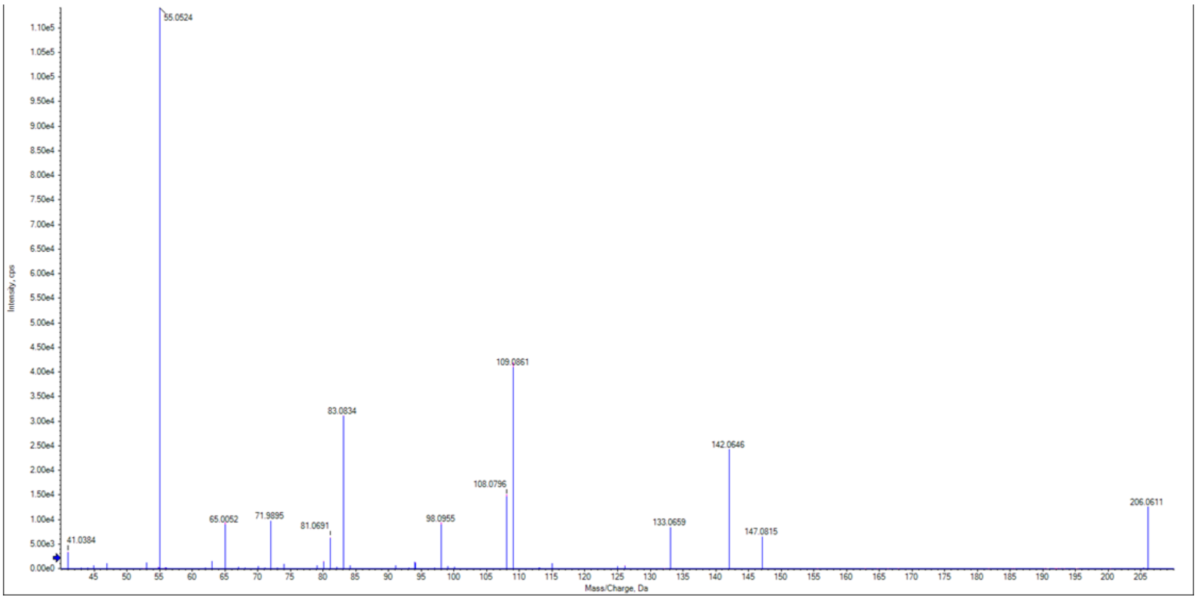


**Figure S5.** MS/MS spectra of *Eutrema japonicum* rhizome sample derived from *n*-hexane/diethyl ether extraction.

**Table S2.** Peak assignment identified the fragmentation pattern of 6-MSITC.

| **Formula** | **Observed (*m*/*z*)** | **Calculated (*m*/*z*)** |
|---|---|---|
| $CH_3SO-(CH_2)_6-NCS$ (M+H) | 206.0611 | 206.0673 |
| $CH_3SO-(CH_2)_5-CH_2^+$ | 147.0815 | 147.0838 |
| $SCN-(CH_2)_5-CH_2^+$ | 142.0646 | 142.0685 |
| $CH_3SO-(CH_2)_4-(CH_2)^+$ | 133.0659 | 133.0683 |
| $CN-(CH_2)_4-(CH)=(CH)^+$ | 108.0796 | 108.0808 |
| $NCS-(CH_2)^+$ | 71.9895 | 71.9902 |

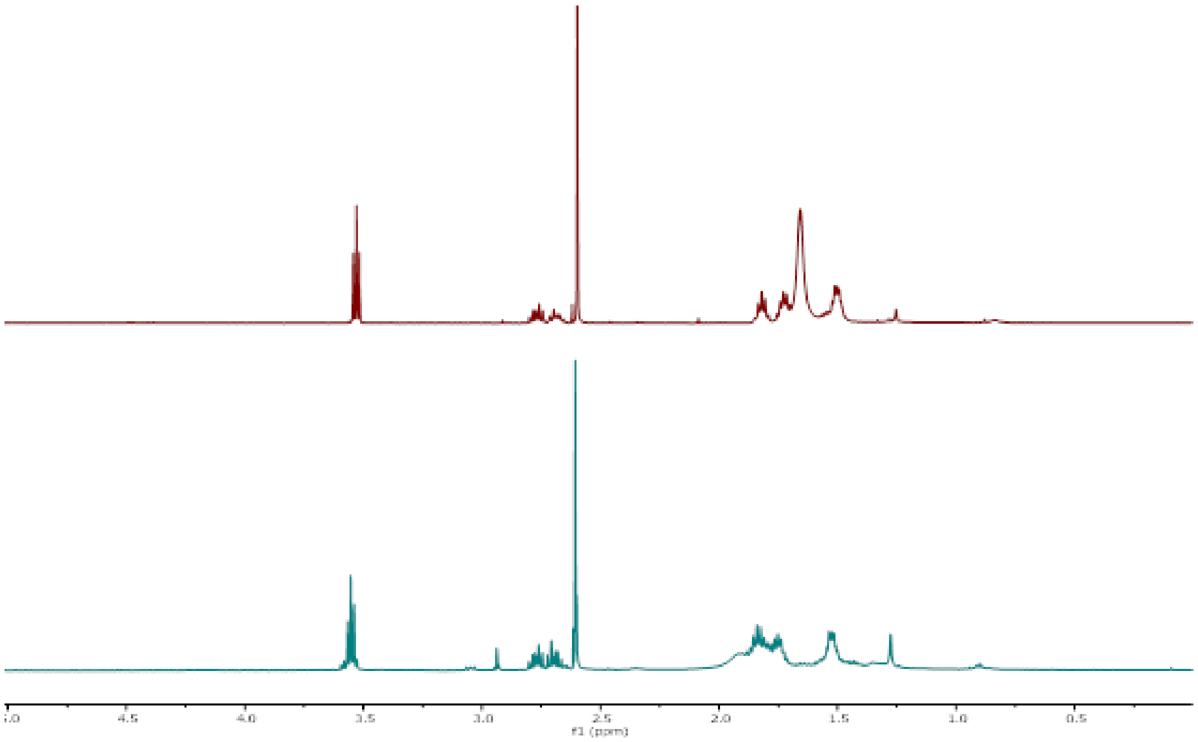


**Figure S6.** $^1H$ NMR comparison for authentic 6-MSITC (Top) and isolated 6-MSITC (bottom) from *E. japonicum* recorded in $CDCl_3$.

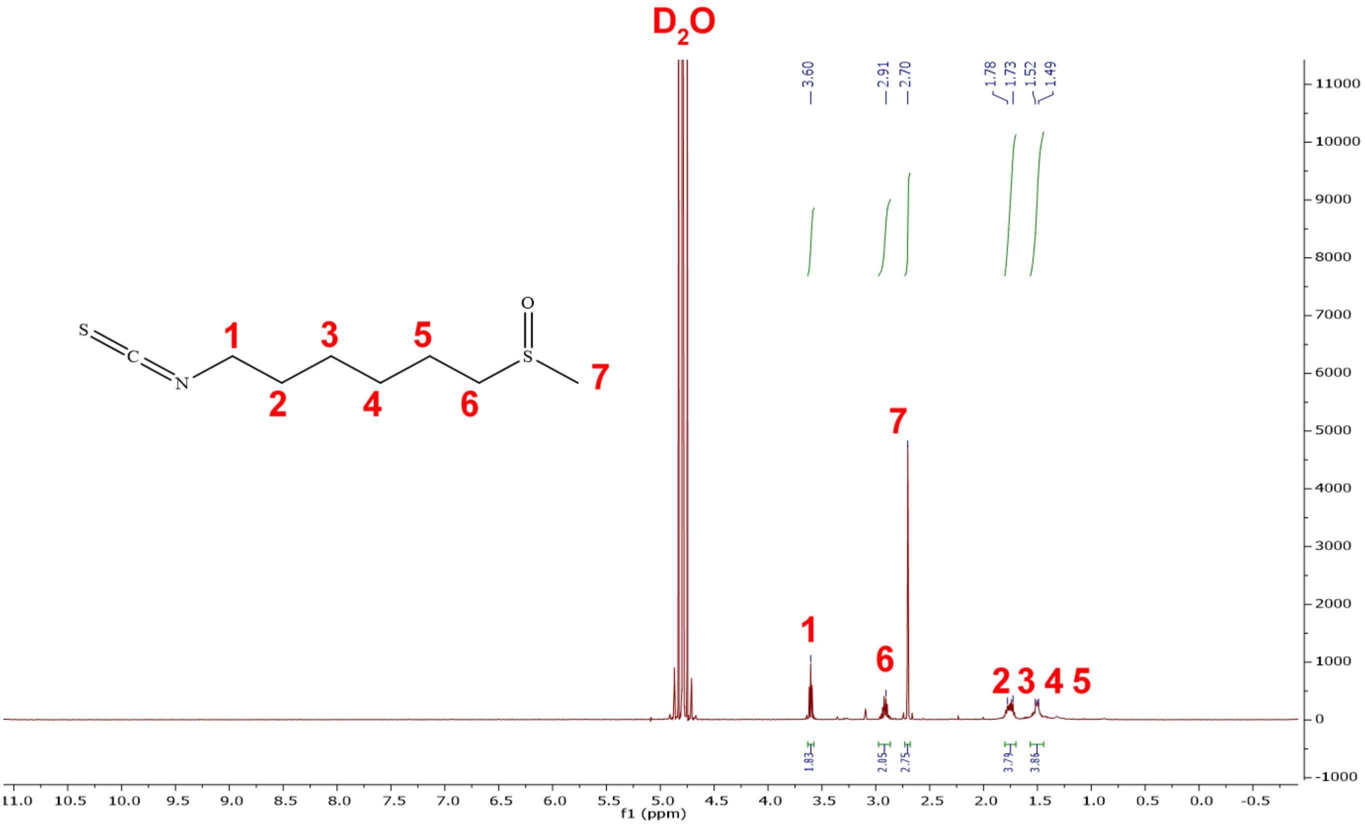


**Figure S7.** The $^{1}$H NMR spectra of 6-MSITC extracted from wasabi rhizome and stems.

**Table S3.** Tabulated $^{1}$H NMR data for 6-MSITC in $D_2O$ at 500 MHz.

| Position | δ[1]H (ppm), multiplicity |
|---|---|
| 1 | 3.60, t |
| 2 | 1.80, m |
| 3 | 1.71, m |
| 4 | 1.55, m |
| 5 | 1.47, m |
| 6 | 2.95, m |
| 7 | 2.88, s |

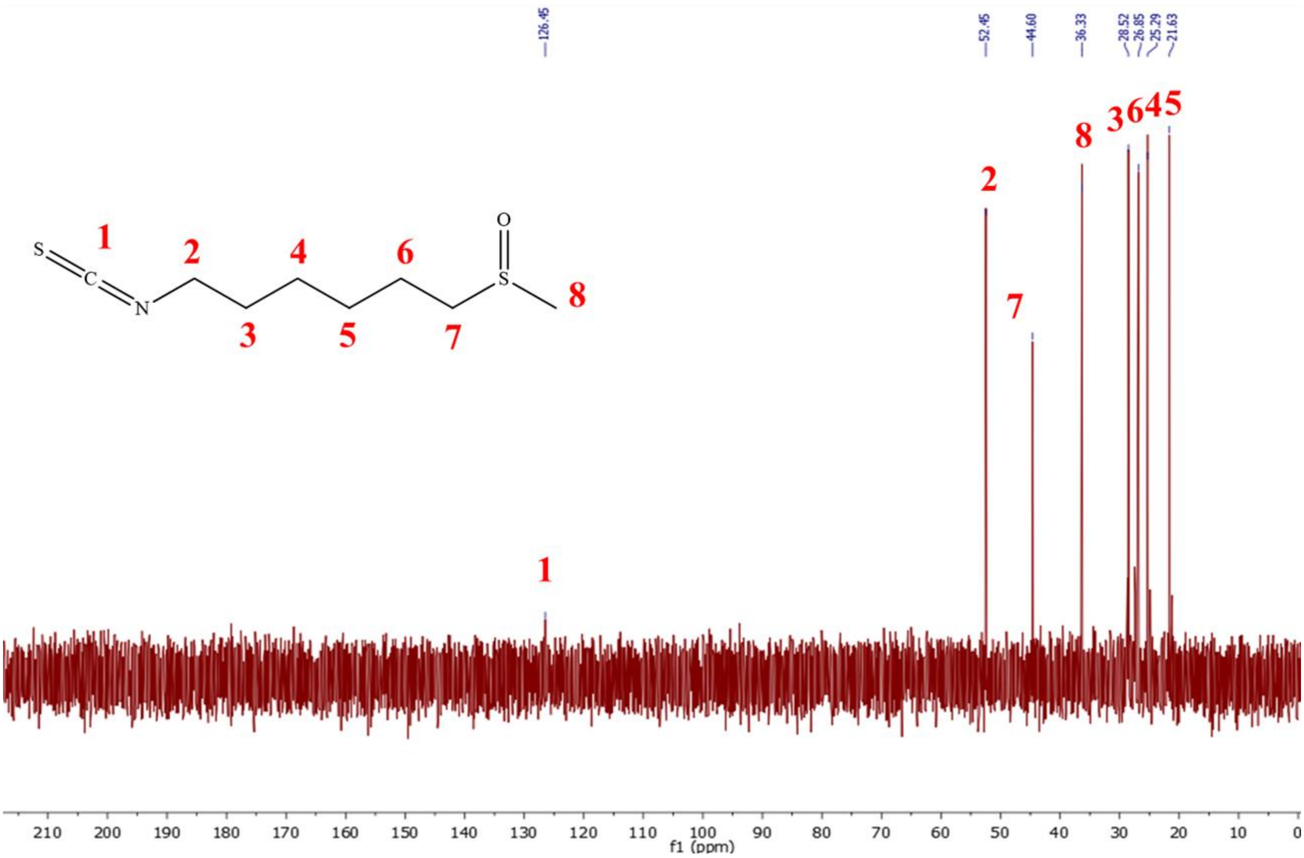


**Figure S8.** $^{13}C$ NMR of the isolated 6-MSITC recorded in $D_2O$ at 125 MHz.

**Table S4.** $^{13}C$ NMR data for 6-MSITC in $D_2O$ at 125 MHz.

| Position | δ$^{13}$C (ppm), type |
|---|---|
| 1 | 126.5, $CH_2$-N |
| 2 | 52.5, $CH_2$ |
| 3 | 28.5, $CH_2$ |
| 4 | 25.3, $CH_2$ |
| 5 | 21.6, $CH_2$ |
| 6 | 26.9, $CH_2$ |
| 7 | 44.6, $CH_2$ |
| 8 | 36.3, $CH_3$-SO- |
